# The prognostic value of IgA anti-citrullinated protein antibodies and rheumatoid factor in an early arthritis population with a treat-to-target approach

**DOI:** 10.1007/s12026-024-09500-w

**Published:** 2024-07-03

**Authors:** Judith W. Heutz, Agnes E. M. Looijen, Jac H. S. A. M. Kuijpers, Marco W. J. Schreurs, Annette H. M. van der Helm-van Mil, Pascal H. P. de Jong

**Affiliations:** 1 Department of Rheumatology, Erasmus Medical Center, Rotterdam, The Netherlands; 2 Department of Immunology, Erasmus Medical Center, Rotterdam, The Netherlands; 3 Department of Rheumatology, Leiden University Medical Center, Leiden, The Netherlands

**Keywords:** IgA autoantibodies, Rheumatoid arthritis, Anti-citrullinated protein antibody, DMARD-free remission

## Abstract

The mucosal origin hypothesis of rheumatoid arthritis has renewed the interest in IgA autoantibodies, but their added value over IgG anti-citrullinated protein antibody (ACPA) and IgM rheumatoid factor (RF) for modern treatment outcomes remains unknown. We aimed to investigate the prognostic value of IgA-ACPA and IgA-RF for treatment outcomes in an early arthritis population. IgA-ACPA/RF isotypes were measured in baseline sera from 480 inflammatory arthritis (IA) patients, who were included in the treatment in the Rotterdam Early Arthritis Cohort trial (tREACH). The tREACH trial was a multicentre, stratified, single-blinded trial with a treat-to-target approach. The prognostic value of IgA-ACPA/RF was determined by evaluating differences in (1) quick-attained (< 6 months after diagnosis) and persistent remission rates, (2) DMARD-free remission and (3) biological use between IA patients with and without IgA-ACPA/RF over 3 years of follow-up. IgA-ACPA was present in 23% of patients and overlapped with IgG-ACPA positivity in 94%. Similarly, IgA-RF overlapped with IgM-RF in 90% of patients. IgA-ACPA positivity was associated with lower DFR rates and more biological use, but this effect was largely mediated by the presence of IgG-ACPA, since this effect disappeared after stratification for IgG-ACPA (HR 0.6, 95%CI 0.2-1.6 for DFR). No differences were observed in ‘quick-attained and persistent remission’ rates and for IgA-RF. Their seems to be no additional value of IgA-ACPA and IgA-RF for modern, long-term clinical outcomes. The effects of IgA-ACPA seen in our study are largely mediated by the presence of IgG-ACPA. Based on these results, there is no rationale for measuring these isotypes in daily practice.

**Supplementary Information:**

The online version contains supplementary material available at 10.1007/s12026-024-09500-w.

## Introduction

The 2022 EULAR research agenda for rheumatoid arthritis (RA) management states that new biomarkers are needed to stratify RA patients and to predict therapeutic response or lack of response [1]. It also emphasises the importance of identification of disease endophenotypes, which is the first step towards personalised medicine [1]. RA patients can be stratified into disease endophenotypes by autoantibodies. IgG anti-citrullinated protein antibody (ACPA) and IgM rheumatoid factor (RF) positivity are used in current RA guidelines as poor prognostic factors for second-line treatment decisions and thus stratify patients into autoantibody positive and negative RA [1]. IgA isotypes of ACPA and RF may help further differentiate RA into endophenotypes.

IgA ACPA and RF have gained renewed interest in the context of the mucosal origin hypothesis in the pathogenesis of RA [2]. Mucosal surfaces have been proposed as the site of initial triggering events, especially in autoantibody positive RA. In this hypothesis, chronic mucosal inflammation transitions to systemic autoimmune disease with loss of the mucosal barrier leading to a systemic instead of local autoantibody response [3]. IgA is the main immunoglobulin isotype produced at mucosal surfaces. If systemically present at high levels, immune complexes can be formed which can damage tissues, including joints [4]. On the other hand, IgA antibodies can be considered anti-inflammatory as they are not strong complement activators and are resistant to proteolytic degradation [3]. Since IgA autoantibodies could thus have opposing pro- and anti-inflammatory roles, their presence could be both positively and negatively associated with disease activity, damage and other treatment outcomes in RA.

In RA, systemic IgA-ACPA and IgA-RF predominantly co-occur with IgG ACPA and IgM RF, although IgA ACPA and IgA RF have also been reported in a proportion of IgG ACPA and IgM RF negative RA patients [5–7]. Two studies looked into the value of IgA ACPA in addition to IgG ACPA. One reported higher disease activity over the course of 3 years in IgA ACPA positive patients, a result that did not remain in the IgG ACPA positive patients only [8]. The other study reported a non-significant higher flare risk in double positive (IgA and IgG ACPA) patients compared to single positive or negative patients [6]. Furthermore, three studies reported on secretory IgA ACPA, which is dimeric instead of monomeric circulating IgA. One investigated the value of secretory IgA ACPA in RA and did not find an association with disease activity or erosive disease [9]. The other did find an association between salivary IgA ACPA and disease activity, but not with erosive disease [7]. The third study found an inverse association between salivary IgA ACPA and erosive disease [10]. Altogether, previous data suggest that IgA ACPA could contribute to worse RA outcomes, but results are inconclusive and the value over IgG ACPA is debatable.

Studies on the prognostic role of IgA-RF report an association of IgA RF with more active and erosive disease [11, 12]. Fortunately, erosive disease is less common nowadays and most studies on the prognostic value of IgA RF took place > 20 years ago. Nowadays, alternative treatment outcomes (as opposed to erosive disease) have become increasingly important, such as sustained remission and DMARD-free remission (DFR) [13]. Previous research already showed that autoantibody positive RA patients are less likely to achieve DFR and more often use biologicals [14–17]. Unfortunately, there is only one study that reported on DFR and other autoantibody isotypes, including IgA. The authors of this study concluded that a higher number of ACPA and RF autoantibody isotypes reduced the chance at achieving DFR [18]. However, this study did not focus on IgA specifically or independently from concomitant IgG presence. Thus, to our knowledge, there are no studies that investigated the added prognostic value of IgA ACPA and IgA RF for the abovementioned ‘modern’ treatment outcomes.

Therefore, to help unravel the current knowledge gap, we determined the added prognostic value of IgA ACPA and IgA RF by looking at the differences in (1) ‘quick-attained and persistent remission’ rates, (2) DFR rates and (3) biological use over a 3-year follow-up period between newly diagnosed inflammatory arthritis patients with and without IgA ACPA and IgA RF who were managed with a treat-to-target approach.

## Methods

### Study population

For this study, inflammatory arthritis (IA) patients who participated in the treatment in the Rotterdam Early Arthritis cohort trial (tREACH) and who had an available baseline serum sample were included [19, 20]. The tREACH was a multicentre, stratified, single-blinded randomised controlled trial. Inclusion criteria for the tREACH were (1) arthritis in ≥ 1 joint, (2) symptom duration < 1 year and (3) age > 18 years. Patients were stratified into 3 groups according to their risk of progressing to persistent arthritis, which was based on the prediction model of Visser [21]. Subsequently, patients were randomised to receive different initial treatment strategies. Patients received one of the following four initial treatment options: (1) triple DMARD therapy (methotrexate (MTX) + sulfasalazine (SASP) + hydroxychloroquine (HCQ) + glucocorticoid (GCs) bridging), (2) MTX with or without GC bridging therapy, (3) HCQ, (4) GC treatment or non-steroidal anti-inflammtory drugs (NSAIDs) (no DMARDs). The tREACH trial had a treat-to-target approach, aiming at low disease activity (DAS < 2.4). Treatment alterations could occur at each 3-monthly visit and treatment was intensified if DAS ≥ 2.4. Intensifications steps were as follows: (1) triple DMARD therapy (MTX + SASP + HCQ), (2) MTX + etanercept, (3) MTX + adalimumab and (4) MTX + abatacept. Medication was tapered if DAS < 1.6 at two consecutive visits. Medication was gradually discontinued, except for HCQ and naproxen, which were immediately stopped. In case of a flare (DAS ≥ 2.4) during tapering, treatment was restarted, according to the stage in the protocol. An extensive description of the study can be found elsewhere [19, 20].

### Autoantibody measurements

At baseline, blood samples were obtained and serum was stored at − 80°. In the baseline sera, the presence of autoantibody isotypes IgG ACPA, IgA ACPA, IgM RF, and IgA RF was determined by automated fluorescence enzyme immunoassay (FEIA) using the Phadia250 EliA™ platform (Thermo Fisher Scientific, Freiburg, Germany), according to the manufacturer’s instruction. These tests have been validated in a group of healthy control subjects by Thermo Fisher (see Supplemental Table S1 for frequency distributions in healthy controls). Cut-offs for autoantibody positivity were employed according to the manufacturer’s instruction. The cut-off levels for autoantibody isotype positivity were ≥ 7 U/ml for IgG ACPA, ≥ 7 U/ml for IgA ACPA, ≥ 3.5 IU/ml for IgM RF and ≥ 14 IU/ml for IgA RF.

### Outcome measures

IgA ACPA and IgA RF positive patients were compared with IgA ACPA and IgA RF negative patients for 3 outcome measures: (1) the proportion of patients that quickly attained (within 6 months) remission (DAS < 1.6) and stayed in remission over a 2-year time period (i.e. ‘quick-attained and persistent remission’), the most favourable outcome in the first years of RA treatment; (2) the proportion of patients that achieved DFR, defined as the absence of clinical synovitis (swollen joints at physical examination) and no DMARD use (including oral glucocorticoids) for ≥ 6 months, over the course of 3 years; and (3) the proportion of patients using a biological over the course of 3 years.

### Statistical analysis

Statistical comparisons of baseline characteristics were made by Student’s *t*-test, *χ*^2^ test, Fisher’s exact test, or Wilcoxon rank-sum test, when appropriate. Baseline characteristics from patients without data on DFR at 3 years—due to lost to follow-up (54%) and due to missing variables (10%)—did not differ (Supplemental Table S2). Differences in the proportion of patients achieving ‘quick-attained and persistent remission’ were analysed using logistic regression models. The probabilities of achieving DFR and biological use over the course of 3 years were visualised with Kaplan Meier curves. Patients that were lost to follow-up were censored. Subsequently, differences in the proportion of patients achieving DFR and using biologicals were analysed using Cox-proportional hazard models. Kaplan Meier curves and analyses were stratified for IgG ACPA and IgM RF, because DFR and biological usage rates are different for autoantibody positive and negative IA patients and because the presence of IgG ACPA and IgM RF influenced the initial treatment strategy, which was partly determined by the risk stratification based on the prediction model for persistent arthritis of Visser et al. [13, 17]. *p* values ≤ 0.05 were considered statistically significant. All statistical analyses were performed in STATA 17.

## Results

### Prevalence of IgA ACPA and IgA RF isotypes

**Fig. 1:**
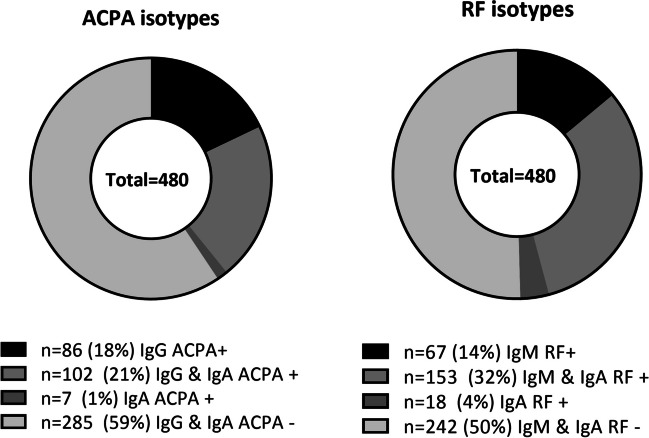
Prevalence of ACPA and RF isotypes. Prevalence of ACPA and RF isotypes in 480 IA patients. Both IgA ACPA and IgA RF predominantly overlap with commonly measured isotypes (IgG ACPA and IgM RF, respectively). Abbreviations: ACPA, anti-citrullinated protein antibody; IA, inflammatory arthritis; RF, rheumatoid factor

Autoantibody isotypes were measured in baseline sera of 480 tREACH patients. A positive IgA ACPA titre was present in 22.7% of IA patients and most of them were also positive for IgG ACPA (overlap of 94%, Fig. 1). Positive IgA RF was present in 35.6% of IA patients, which overlapped with IgM RF in 90% of patients (Fig. 1). Only a few IA patients were solely positive for IgA ACPA or IgA RF (1% and 4%, respectively).

### Study population

Baseline characteristics of the 480 included patients are stratified for IgA ACPA and RF presence or absence (Table 1). IgA isotype positive IA patients had higher disease activity and inflammatory markers at baseline compared to IgA isotype negative IA patients, both for IgA ACPA and IgA RF positivity.

**Table 1:** Baseline characteristics of included IA patients (*n* = 480), stratified for IgA ACPA or RF presence or absence

	IgA ACPA +		IgA ACPA −		*p value*	IgA RF +		IgA RF −		*p value*
	*n* = 109		*n* = 371			*n* = 171		*n* = 309		
*Gender, female, n (%)*	67	(61)	252	(68)	*0.21*	110	(64)	209	(68)	*0.46*
*Age, mean (sd)*	55	(13)	52	(15)	*0.06*	54	(13)	52	(15)	*0.20*
*Symptom duration (weeks), median (IQR)*	22	(13–30)	21	(13–31)	*0.88*	21	(13–30)	21	(13–32)	*0.64*
*DAS44, mean (sd)*	3.3	(1)	3.0	(1)	*0.01*	3.3	(1)	3.0	(1)	*0.006*
*Swollen joint count, median (IQR)*	8	(4–12)	5	(2–9)	< *0.001*	8	(4–12)	4	(2–8)	< *0.001*
*Tender joint count, median (IQR)*	8	(3–14)	7	(3–13)	*0.71*	7	(3–14)	7	(4–13)	*0.96*
*1987/2010 RA criteria, n (%)*	101	(93)	234	(63)	< *0.001*	158	(92)	177	(57)	< *0.001*
*CRP (mg/l), median (IQR)*	10	(5–22)	6	(3–17)	*0.009*	9	(4–24)	6	(3–15)	*0.002*
*ESR (mm/h), median (IQR)*	25	(15–42)	16	(8–30)	< *0.001*	24	(14–42)	15	(8–29)	< *0.001*
*IgG ACPA* + *, n (%)*	102	(94)	86	(23)	< *0.001*	149	(87)	39	(13)	< *0.001*
IgG ACPA level (IU/ml), median (IQR)*	340	(327–340)	96	(36–189)	< *0.001*	327	(119–340)	175	(55–340)	*0.09*
*IgM RF* + *, n (%)*	99	(91)	121	(33)	< *0.001*	153	(89)	67	(22)	< *0.001*
IgM RF level (IU/ml), median (IQR)**	81	(30–170)	23	(9–52)	< *0.001*	63	(29–135)	10	(5–48)	< *0.001*

*ACPA* anti-citrullinated protein antibody, *CRP* C-reactive protein, *DAS* disease activity score, *ESR* erythrocyte sedimentation rate, *IA* inflammatory arthritis, *IQR* interquartile range, *RA* rheumatoid arthritis, *RF* rheumatoid factor, *sd* standard deviation

*In IgG ACPA positive patients

**In IgM RF positive patients

### Quick-persistent remission

**Fig. 2:**
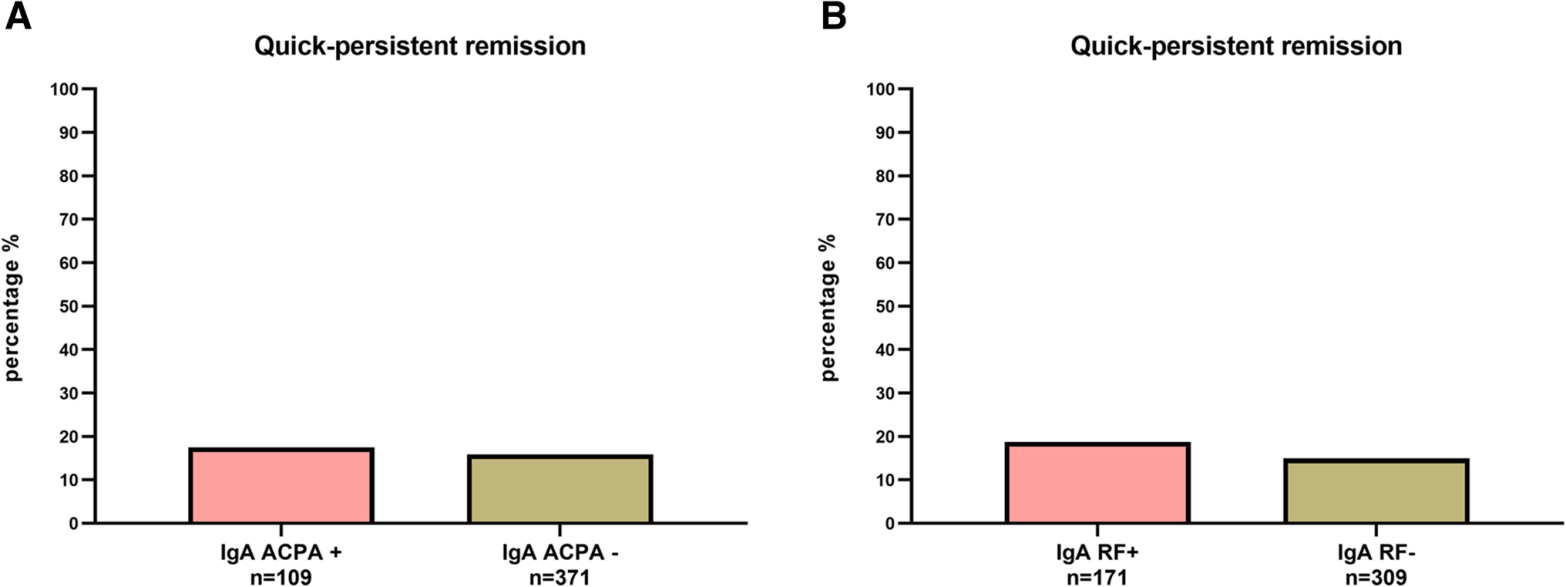
Quick-persistent remission in IgA ACPA/RF positive versus IgA ACPA/RF negative patients. ‘Quick-attained and persistent’ remission rates in **A** IgA ACPA positive vs. negative patients and in **B** IgA RF positive vs. negative patients. ‘Quick-attained and persistent’ remission was defined as the proportion of patients that quickly attained (within 6 months) remission (DAS < 1.6) and stayed in remission until 2 years of follow-up. Data stratified for commonly measured isotypes (IgG ACPA and IgM RF) showed similar results (Supplemental Fig. S1). Abbreviations: ACPA, anti-citrullinated protein antibody; RF, rheumatoid factor

Sixteen percent of IA patients achieved remission within 6 months that persisted over 2 years. These ‘quick-attained and persistent’ remission rates did not significantly differ between IgA ACPA positive and negative patients (OR 1.1 (95% CI 0.6–2.0), Fig. 2) and IgA RF positive and negative patients (OR 1.3 (95% CI 0.8–2.2), Fig. 2). Stratified analysis for commonly measured isotypes (IgG ACPA and IgM RF) showed similar results (Supplemental Fig. S1).

### IgA ACPA and the chance of DFR and biological use

**Fig. 3:**
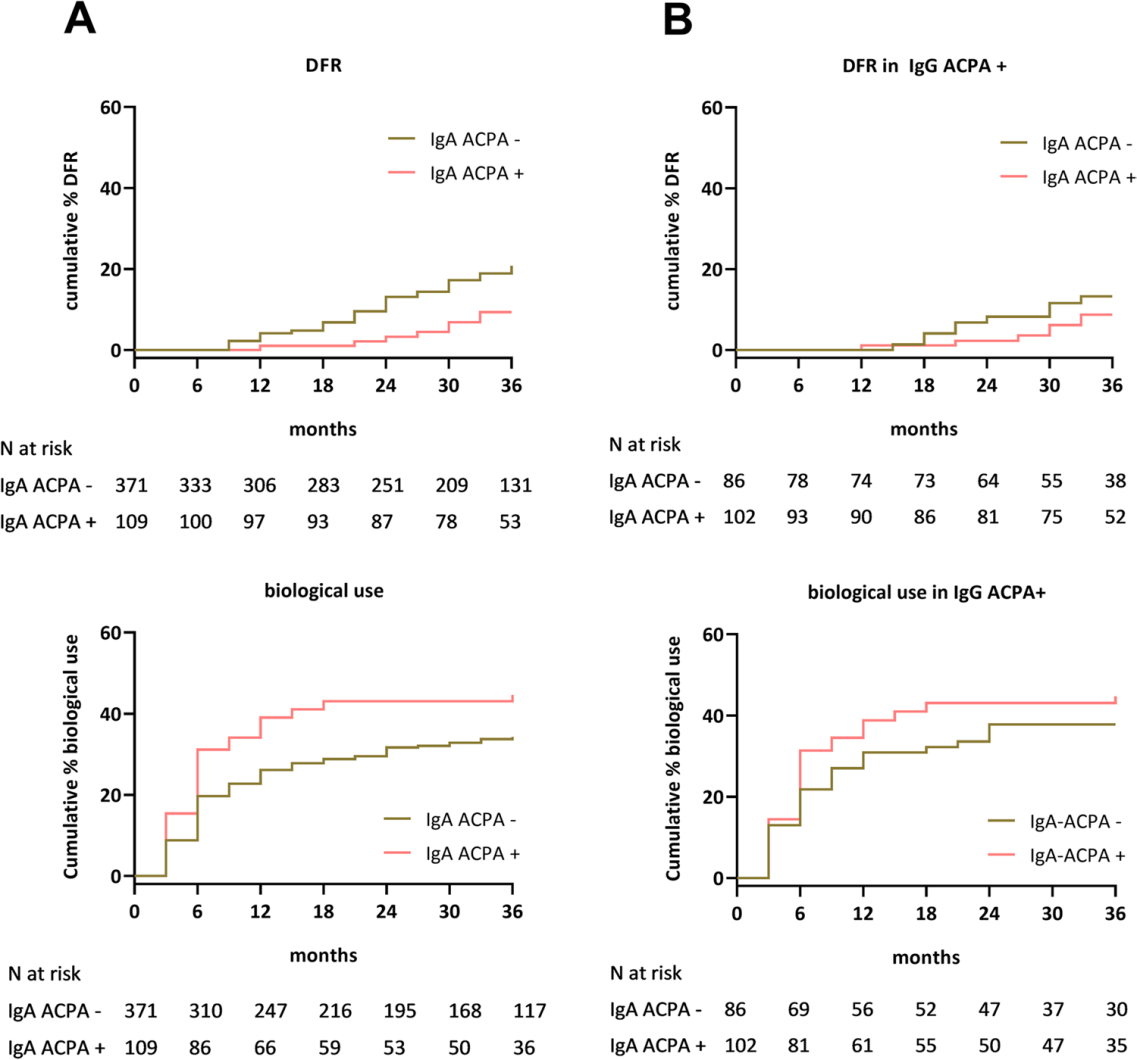
DMARD-free remission and biological use in IgA ACPA positive vs. IgA ACPA negative patients. Kaplan Meier curves for achievement of DMARD-free remission and biological use over 36 months in **A** the whole population and in **B** IgG ACPA positive patients comparing IgA ACPA positive and IgA ACPA negative patients. Abbreviation: ACPA, anti-citrullinated protein antibody

IgA ACPA positive patients had a significantly lower chance at achieving DFR over 3 years compared to IgA ACPA negative patients (cumulative percentage 9.4% vs. 20.8%, HR 0.41, 95% CI 0.20–0.86, Fig. 3A). When adjusting for the effect of IgG ACPA, the analysis still revealed lower DFR rates for IgA ACPA positive patients compared to IgA ACPA negative patients, but this finding was not significant anymore (cumulative percentage 8.7% vs. 13.3%, HR 0.60, 95% CI 0.22–1.61, Fig. 3B). Furthermore, IgA ACPA positive patients had a significantly higher chance at biological use over 3 years compared to IgA ACPA negative patients (cumulative percentage 44.6% vs. 34.3%, HR 1.44, 95% CI 1.02–2.04, Fig. 3A). After adjustment for IgG ACPA positivity, biological use was still numerically higher in IgA ACPA positive patients, but this finding did not remain significant (cumulative percentage 44.7% vs. 37.8%, HR 1.24, 95% CI 0.77–1.98, Fig. 3B).

### IgA RF and the chance of DFR and biological use

**Fig. 4:**
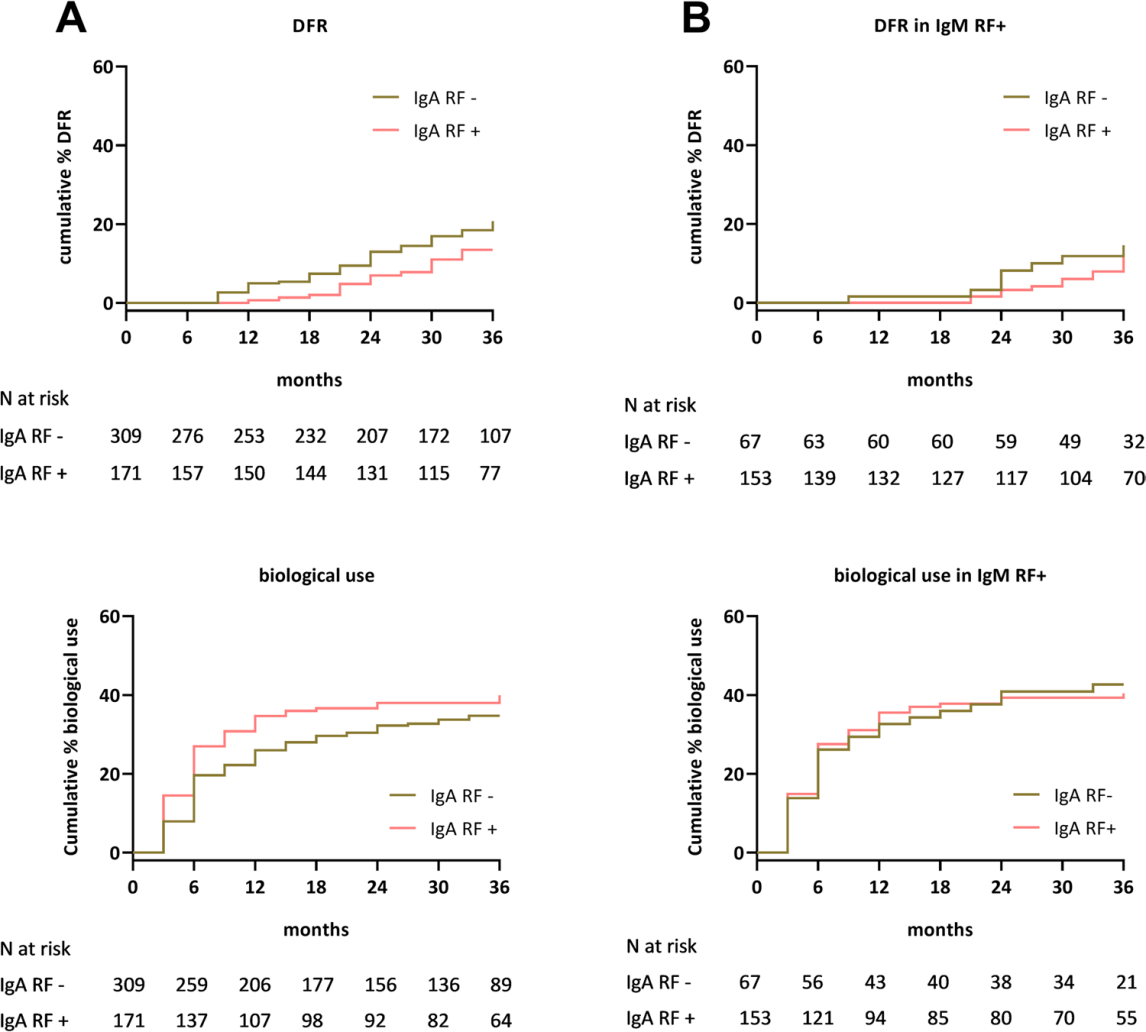
DMARD-free remission and biological use in IgA RF positive vs. IgA RF negative patients. Kaplan Meier curves for achievement of DMARD-free remission and biological use over 36 months in **A** the whole population and in **B** IgM-RF positive patients comparing IgA RF positive and IgA RF negative patients. Abbreviation: RF, rheumatoid factor

IgA RF positive patients had a non-significant lower chance at achieving DFR over 3 years compared to IgA RF negative patients (cumulative percentage 13.5% vs. 20.7%, HR 0.62, 95% CI 0.36–1.06, Fig. 4A). This numerical difference disappeared after taking IgM RF positivity into account (cumulative percentage 14.5% vs. 14.6%, HR 0.84, 95% CI 0.35–2.00, Fig. 4B). In addition, the chance of biological use was similar for IgA RF positive and IgA RF negative patients, both in the whole population (cumulative percentage 39.9% vs. 34.7%, HR 1.24, 95% CI 0.90–1.70, Fig. 4A) and after accounting for IgM RF positivity (cumulative percentage 40.4% vs. 42.7%, HR 0.96, 95% CI 0.61–1.51, Fig. 4B).

## Discussion

In this study, we aimed to evaluate the prognostic value of the IgA autoantibody isotypes of ACPA and RF in an early arthritis population. We showed that positivity for both IgA ACPA and IgA RF almost completely overlapped with positivity for the commonly measured autoantibody isotypes IgG ACPA and IgM RF. The prognostic value of IgA ACPA and IgA RF was studied for the outcomes of ‘quick-attained and persistent’ remission rates, the probability of reaching DFR, and the probability of using a biological over the course of 3 years. Both IgA ACPA and IgA RF were not associated with ‘quick-attained and persistent remission’ rates. IgA-ACPA was significantly associated with DFR achievement and the risk of biological use over 3 years. However, most of this risk should be appointed to IgG-ACPA positivity in that group, since the same analysis in IgG-ACPA positive patients did not show a significant difference in DFR and biological use. For IgA RF, there was no significant difference between IgA RF positive and negative patients in achievement of DFR and risk of biological use.

Our finding that IgA isotypes have a large overlap with commonly measured isotypes is in line with previous studies. A previous study in early RA showed that the majority of IgA ACPA and/or IgA RF positive patients were also positive for IgG ACPA and/or IgM RF [22]. One study in early and established RA patients found that 5.2% of the IgG ACPA and IgM RF negative population was positive for IgA ACPA and/or IgA RF, which is similar to our data (1% IgA ACPA positivity and 4% IgM RF positivity) [5]. Another study found a 2.5–5.8% positivity for IgA RF in the IgM RF negative RA population [23]. Altogether, only a very small number of early arthritis patients without commonly measured autoantibodies are positive for IgA ACPA and IgA RF.

The measurement of IgA ACPA and IgA RF in a clinical setting is only relevant when they can provide prognostic information for RA patients for modern treatment outcomes and when they have added value over the commonly measured antibodies. Therefore, we studied the prognostic value of IgA ACPA and IgA RF for the outcomes of ‘quick-attained and persistent’ remission, DFR and biological use. Regarding ACPA, it is well known that the IgG isotype is of prognostic value [17, 24, 25]. Previous literature has shown that chances of achieving DFR are lower and the risk of biological use is higher in IgG ACPA positive patients compared to IgG ACPA negative patients [14, 24, 26]. Our results showed that IgA ACPA was associated with lower DFR rates and more biological use, but this was largely mediated by the known effect of IgG ACPA since most patients were also IgG-ACPA positive and when accounting for IgG ACPA positivity these differences mostly disappeared.

In our data, IgA RF was not associated with any of the aforementioned disease outcomes. Previous studies that investigated the relationship between IgA RF positivity and disease outcomes have shown that IgA RF positivity is associated with more active and erosive disease [11, 27–32]. However, most of these studies come from years when IgG ACPA was not yet known as the best predictive autoantibody for treatment outcomes in RA and, therefore, information on IgG ACPA in these studies is lacking [11, 28–32]. A more recent study that included information on ACPA also found that IgA-RF was a predictive factor for erosive disease, but also showed that IgG ACPA in combination with IgM RF and not IgA RF predicted erosive disease more accurate [33]. All aforementioned studies focused on erosive disease (and/or disease activity), which is a much less common feature of RA nowadays due to better treatment options. To our knowledge, the added value of IgA RF for modern treatment outcomes such as DFR and biological use has not been evaluated in past and current literature and we are the first to report on this.

Our study is relevant in the context of the mucosal origin hypothesis, in which IgA autoantibodies might play a role in triggering events that lead to RA [2, 3]. Based on our findings, we believe that a subgroup of patients with IgA autoantibodies and thus possibly a specific underlying trigger for their disease do not necessarily have a different prognosis compared to IgA negative patients. An initial disease trigger that in this case might be reflected by the presence of circulating IgA autoantibodies does not necessarily imply a different clinical course in a later disease stage. Nevertheless, reporting these results is important because clear markers for prognosis are still lacking and research aimed at finding prognostic biomarkers is needed for precision medicine [34]. Alternatively, circulating IgA ACPA does not unquestionably reflect an initial disease trigger at the mucosal site, since there is evidence that circulating IgA does not correlate with salivary IgA presence in RA [10]. Circulating IgA antibodies might be produced independently of mucosal IgA and reflect more of a general broad autoantibody profile caused by more humoral autoimmunity. The numerical difference in DFR and biological use in our data between IgA positive and negative patients in the IgG ACPA positive group could be a result of a stronger humoral autoimmunity and consequently lead to a more severe disease with worse outcomes. This is also in line with what Moel et al. reported, who showed that a broader autoantibody profile, possibly caused by more humoral immunity, is associated with worse treatment outcomes in early RA [18].

Limitations of the current study include the small number of events for DFR, which gave less power to find statistical significance for some numerical differences that we saw in the data. In addition, correcting for confounders was not possible due to the small number of events. However, the findings for biological use were concordant with the findings for DFR, and due to this consistency, we believe our results are valid. Secondly, the unstratified analyses might have been influenced not only by the concomitant presence of IgG ACPA and IgM RF but also by differences in the initial treatment strategy, since these were IgG ACPA and IgM RF dependent [21]. Thus, the effect of IgA ACPA on DFR and biological use might have been mediated not only by the presence of IgG ACPA but also by the initial treatment strategy. Moreover, there was a relatively high percentage of missing data (the highest being 64% for DFR at 3 years of follow-up), mainly due to lost to follow-up (54% and 10% for missing variables of patients still in follow-up). Baseline characteristics between patients with data on DFR at 3 years did not remarkably differ, which reassured us that our results were not biased by differences between patients that were and were not lost to follow-up. Finally, our study was executed in a treat-to-target setting, in which treatment was intensified and tapered according to a fixed medication protocol. Although a treat-to target management approach is recommended for RA, this protocolized treatment regimen might not be completely generalizable to a real-life setting, in which treatment decisions are also based on the perspective of the treating rheumatologist and patient.

## Conclusion

The presence of IgA ACPA and IgA RF almost completely overlaps with the presence of the commonly measured isotypes IgG ACPA and IgM RF. In addition, both presence of IgA ACPA and IgA RF seems to have no additional value over concomitant IgG ACPA and IgM RF presence for the outcomes of ‘quick-attained and persistent’ remission, achievement of DFR and biological use in an early arthritis population with a treat-to-target approach. The small numerical difference for IgA positive patients for these outcomes in the IgG ACPA positive group could be a reflection of a stronger humoral autoimmunity causing a slightly worse disease course in these patients. To conclude, there seems to be no rationale for measuring IgA ACPA and IgA RF in daily clinical practice.

## Supplementary Information

Below is the link to the electronic supplementary material.

Supplementary file1 (DOCX 41 kb)

## Data Availability

The data used in this study are available from the corresponding author upon reasonable request.

## Abbreviations

RA: Rheumatoid arthritis
ACPA: Anti-citrullinated protein antibody
RF: Rheumatoid factor
IA: Inflammatory arthritis
DFR: DMARD-free remission
tREACH trial: Treatment in the Rotterdam Early Arthritis Cohort trial
MTX: Methotrexate
SASP: Sulfasalazine
HCQ: Hydroxychloroquine
GC: Glucocorticoid
DAS: Disease activity score
FEIA: Fluorescence enzyme immunoassay

## References

[1] Smolen JS, Landewé RBM, Bergstra SA, Kerschbaumer A, Sepriano A, Aletaha D, et al. EULAR recommendations for the management of rheumatoid arthritis with synthetic and biological disease-modifying antirheumatic drugs: 2022 update. Ann Rheum Dis. 2023;82(1):3–18. 36357155 10.1136/ard-2022-223356

[2] Derksen V, Allaart CF, Van der Helm-Van Mil AHM, Huizinga TWJ, Toes REM, van der Woude D. In rheumatoid arthritis patients, total IgA1 and IgA2 levels are elevated: implications for the mucosal origin hypothesis. Rheumatology (Oxford). 2022;62(1):407–16. 35416963 10.1093/rheumatology/keac237 PMC9788813

[3] Holers VM, Demoruelle MK, Kuhn KA, Buckner JH, Robinson WH, Okamoto Y, et al. Rheumatoid arthritis and the mucosal origins hypothesis: protection turns to destruction. Nat Rev Rheumatol. 2018;14(9):542–57. 30111803 10.1038/s41584-018-0070-0 PMC6704378

[4] Aleyd E, Al M, Tuk CW, van der Laken CJ, van Egmond M. IgA complexes in plasma and synovial fluid of patients with rheumatoid arthritis induce neutrophil extracellular traps via FcαRI. J Immunol. 2016;197(12):4552–9. 27913645 10.4049/jimmunol.1502353

[5] Sieghart D, Platzer A, Studenic P, Alasti F, Grundhuber M, Swiniarski S, et al. Determination of autoantibody isotypes increases the sensitivity of serodiagnostics in rheumatoid arthritis. Front Immunol. 2018;9:876. 29740454 10.3389/fimmu.2018.00876 PMC5929149

[6] Sokolova MV, Hagen M, Bang H, Schett G, Rech J, Steffen U, et al. IgA anti-citrullinated protein antibodies are associated with flares during DMARD tapering in rheumatoid arthritis. Rheumatology (Oxford). 2022;61(5):2124–31. 34508547 10.1093/rheumatology/keab585

[7] Roos Ljungberg K, Börjesson E, Martinsson K, Wetterö J, Kastbom A, Svärd A. Presence of salivary IgA anti-citrullinated protein antibodies associate with higher disease activity in patients with rheumatoid arthritis. Arthritis Res Ther. 2020;22(1):274. 33225988 10.1186/s13075-020-02363-0 PMC7681967

[8] Svärd A, Kastbom A, Reckner-Olsson A, Skogh T. Presence and utility of IgA-class antibodies to cyclic citrullinated peptides in early rheumatoid arthritis: the Swedish TIRA project. Arthritis Res Ther. 2008;10(4):R75. 18601717 10.1186/ar2449 PMC2575621

[9] Roos K, Martinsson K, Ziegelasch M, Sommarin Y, Svärd A, Skogh T, et al. Circulating secretory IgA antibodies against cyclic citrullinated peptides in early rheumatoid arthritis associate with inflammatory activity and smoking. Arthritis Res Ther. 2016;18(1):119. 27215344 10.1186/s13075-016-1014-1 PMC4877943

[10] Svärd A, Kastbom A, Sommarin Y, Skogh T. Salivary IgA antibodies to cyclic citrullinated peptides (CCP) in rheumatoid arthritis. Immunobiology. 2013;218(2):232–7. 22652412 10.1016/j.imbio.2012.04.011

[11] Teitsson I, Withrington RH, Seifert MH, Valdimarsson H. Prospective study of early rheumatoid arthritis. I. Prognostic value of IgA rheumatoid factor. Ann Rheum Dis. 1984;43(5):673–8. 6497459 10.1136/ard.43.5.673 PMC1001507

[12] Withrington RH, Teitsson I, Valdimarsson H, Seifert MH. Prospective study of early rheumatoid arthritis. II. Association of rheumatoid factor isotypes with fluctuations in disease activity. Ann Rheum Dis. 1984;43(5):679–85. 6497460 10.1136/ard.43.5.679 PMC1001508

[13] Verstappen M, van Mulligen E, de Jong PHP, van der Helm-Van Mil AHM. DMARD-free remission as novel treatment target in rheumatoid arthritis: a systematic literature review of achievability and sustainability. RMD Open. 2020;6(1):e001220. 10.1136/rmdopen-2020-001220 PMC7299506 32393523

[14] van der Woude D, Young A, Jayakumar K, Mertens BJ, Toes RE, van der Heijde D, et al. Prevalence of and predictive factors for sustained disease-modifying antirheumatic drug-free remission in rheumatoid arthritis: results from two large early arthritis cohorts. Arthritis Rheum. 2009;60(8):2262–71. 19644846 10.1002/art.24661

[15] van der Kooij SM, Goekoop-Ruiterman YP, de Vries-Bouwstra JK, Güler-Yüksel M, Zwinderman AH, Kerstens PJ, et al. Drug-free remission, functioning and radiographic damage after 4 years of response-driven treatment in patients with recent-onset rheumatoid arthritis. Ann Rheum Dis. 2009;68(6):914–21. 18662933 10.1136/ard.2008.092254

[16] Heimans L, Akdemir G, Boer KV, Goekoop-Ruiterman YP, Molenaar ET, van Groenendael JH, et al. Two-year results of disease activity score (DAS)-remission-steered treatment strategies aiming at drug-free remission in early arthritis patients (the IMPROVED-study). Arthritis Res Ther. 2016;18:23. 26794605 10.1186/s13075-015-0912-y PMC4721018

[17] Luurssen-Masurel N, van Mulligen E, Weel-Koenders A, Hazes JMW, de Jong PHP. The susceptibility of attaining and maintaining DMARD-free remission in different (rheumatoid) arthritis phenotypes. Rheumatology (Oxford). 2021;5:keab631. 10.1093/rheumatology/keab631 34352094

[18] de Moel EC, Derksen V, Stoeken G, Trouw LA, Bang H, Goekoop RJ, et al. Baseline autoantibody profile in rheumatoid arthritis is associated with early treatment response but not long-term outcomes. Arthritis Res Ther. 2018;20(1):33. 29482627 10.1186/s13075-018-1520-4 PMC5828136

[19] Claessen SJ, Hazes JM, Huisman MA, van Zeben D, Luime JJ, Weel AE. Use of risk stratification to target therapies in patients with recent onset arthritis; design of a prospective randomized multicenter controlled trial. BMC Musculoskelet Disord. 2009;10:71. 19538718 10.1186/1471-2474-10-71 PMC2702315

[20] de Jong PH, Hazes JM, Han HK, Huisman M, van Zeben D, van der Lubbe PA, et al. Randomised comparison of initial triple DMARD therapy with methotrexate monotherapy in combination with low-dose glucocorticoid bridging therapy; 1-year data of the tREACH trial. Ann Rheum Dis. 2014;73(7):1331–9. 24788619 10.1136/annrheumdis-2013-204788 PMC4078755

[21] Visser H, le Cessie S, Vos K, Breedveld FC, Hazes JM. How to diagnose rheumatoid arthritis early: a prediction model for persistent (erosive) arthritis. Arthritis Rheum. 2002;46(2):357–65. 11840437 10.1002/art.10117

[22] Nell-Duxneuner V, Machold K, Stamm T, Eberl G, Heinzl H, Hoefler E, et al. Autoantibody profiling in patients with very early rheumatoid arthritis: a follow-up study. Ann Rheum Dis. 2010;69(1):169–74. 19153104 10.1136/ard.2008.100677

[23] Van Hoovels L, Vander Cruyssen B, Sieghart D, Bonroy C, Nagy E, Pullerits R, et al. IgA rheumatoid factor in rheumatoid arthritis. Clin Chem Lab Med. 2022;60(10):1617–26. 35790193 10.1515/cclm-2022-0244

[24] Matthijssen XME, Niemantsverdriet E, Huizinga TWJ, van der Helm-van Mil AHM. Enhanced treatment strategies and distinct disease outcomes among autoantibody-positive and -negative rheumatoid arthritis patients over 25 years: a longitudinal cohort study in the Netherlands. PLoS Med. 2020;17(9):e1003296. 32960885 10.1371/journal.pmed.1003296 PMC7508377

[25] Luurssen-Masurel N, Weel A, Hazes J, de Jong P, t Rgi. The impact of different (rheumatoid) arthritis phenotypes on patients’ lives. Rheumatology (Oxford). 2021;60(8):3716–26. 10.1093/rheumatology/keaa845 PMC8328508 33237330

[26] van der Woude D, Visser K, Klarenbeek NB, Ronday HK, Peeters AJ, Kerstens PJ, et al. Sustained drug-free remission in rheumatoid arthritis after DAS-driven or non-DAS-driven therapy: a comparison of two cohort studies. Rheumatology (Oxford). 2012;51(6):1120–8. 22337939 10.1093/rheumatology/ker516

[27] Ateş A, Kinikli G, Turgay M, Akay G, Tokgöz G. Effects of rheumatoid factor isotypes on disease activity and severity in patients with rheumatoid arthritis: a comparative study. Clin Rheumatol. 2007;26(4):538–45. 16804738 10.1007/s10067-006-0343-x

[28] Houssien DA, Jónsson T, Davies E, Scott DL. Rheumatoid factor isotypes, disease activity and the outcome of rheumatoid arthritis: comparative effects of different antigens. Scand J Rheumatol. 1998;27(1):46–53. 9506878 10.1080/030097498441173

[29] Tarkowski A, Nilsson LA. Isotype-specific measurement of rheumatoid factor with reference to clinical features of rheumatoid arthritis. J Clin Lab Immunol. 1983;12(3):129–35. 6663605

[30] Eggelmeijer F, Otten HG, de Rooy HH, Daha MR, Breedveld FC. Significance of rheumatoid factor isotypes in seronegative rheumatoid arthritis. Rheumatol Int. 1990;10(1):43–6. 2353153 10.1007/BF02274780

[31] Winska Wiloch H, Thompson K, Young A, Corbett M, Shipley M, Hay F. IgA and IgM rheumatoid factors as markers of later erosive changes in rheumatoid arthritis (RA). Scand J Rheumatol Suppl. 1988;75:238–43. 3238356 10.3109/03009748809096770

[32] van Zeben D, Hazes JM, Zwinderman AH, Cats A, van der Voort EA, Breedveld FC. Clinical significance of rheumatoid factors in early rheumatoid arthritis: results of a follow up study. Ann Rheum Dis. 1992;51(9):1029–35. 1417131 10.1136/ard.51.9.1029 PMC1004831

[33] Vencovský J, Machácek S, Sedová L, Kafková J, Gatterová J, Pesáková V, et al. Autoantibodies can be prognostic markers of an erosive disease in early rheumatoid arthritis. Ann Rheum Dis. 2003;62(5):427–30. 12695154 10.1136/ard.62.5.427 PMC1754544

[34] Aletaha D. Precision medicine and management of rheumatoid arthritis. J Autoimmun. 2020;110:102405. 32276742 10.1016/j.jaut.2020.102405

