# Supplementary material for: The prognostic value of IgA anti-citrullinated protein antibodies and rheumatoid factor in an early arthritis population with a treat-to-target approach
Source: Immunol Res. 2024 Jul 3;72(5):982–90. doi: 10.1007/s12026-024-09500-w (PMC11564213; doi:10.1007/s12026-024-09500-w)
Supplement: Supplementary file 1 — Supplementary file1 (DOCX 41 kb) [file 12026_2024_9500_MOESM1_ESM.docx]

**Supplemental material**

| **Supplemental Table S1.** Frequency distributions from the Phadia250 EliA obtained from healthy subjects | | | | | |
| --- | --- | --- | --- | --- | --- |
| Test | Unit | No. of samples | Mean Value | 95% percentile | 99% percentile |
| EliA IgG ACPA | U/ml | 400 | 2.6 | 4.3 | 6.2 |
| EliA IgA ACPA | U/ml | 400 | 2.4 | 5.1 | 6.7 |
| EliA IgM RF | IU/ml | 400 | 1.5 | 4.9 | 17 |
| EliA IgA RF | IU/ml | 400 | 3.3 | 8.6 | 18 |
| Adapted from: Directions for Use manuals per test available at [Allergy and Autoimmune Diagnostics Product Catalog \| Thermo Fisher Scientific](https://eur01.safelinks.protection.outlook.com/?url=https%3A%2F%2Fwww.thermofisher.com%2Fphadia%2Fwo%2Fen%2Fproduct-catalog.html%3Fsolution%3DEliA%26region%3DNL&data=05%7C02%7Cj.heutz%40erasmusmc.nl%7Cc8934000b7214ee890b208dc6dd2a97d%7C526638ba6af34b0fa532a1a511f4ac80%7C0%7C0%7C638505998759784894%7CUnknown%7CTWFpbGZsb3d8eyJWIjoiMC4wLjAwMDAiLCJQIjoiV2luMzIiLCJBTiI6Ik1haWwiLCJXVCI6Mn0%3D%7C0%7C%7C%7C&sdata=AnkRThE3Sa9FeTv1iCDEKNCSwIhsgspwpWztevHYzqc%3D&reserved=0)  Abbreviations: ACPA, anti-citrullinated protein antibody; RF, rheumatoid factor | | | | | |

| **Supplemental Table S2**. Baseline characteristics of patients with DFR data at 3 years of follow up vs. patients without data on DFR at 3 years. Missing data was due to lost to follow up (~54%) or missing variables* (~10%) | | | | |
| --- | --- | --- | --- | --- |
|  | With DFR data | | Without DFR data | |
|  | N=173 | | N=307 | |
| *Gender, female, n (%)* | 110 | (64) | 209 | (68) |
| *Age, mean (sd)* | 53 | (14) | 53 | (15) |
| *Symptom duration (weeks), median (IQR)* | 21 | (14-30) | 20 | (13-31) |
| *DAS44, mean (sd)* | 3.1 | (1) | 3.1 | (1) |
| *Swollen joint count, median (IQR)* | 6 | (3-11) | 5 | (2-10) |
| *Tender joint count, median (IQR)* | 7 | (3-12) | 8 | (3-14) |
| *1987/2010 RA criteria, n (%)* | 123 | (71) | 212 | (69) |
| *CRP (mg/l), median (IQR)* | 7 | (4-18) | 7 | (3-18) |
| *ESR (mm/h), median (IQR)* | 19 | (10-34) | 17 | (10-35) |
| *IgA ACPA +, n (%)* | 40 | (23) | 69 | (22) |
| *IgG ACPA+, n (%)* | 72 | (41) | 116 | (38) |
| *IgA RF +, n (%)* | 66 | (38) | 105 | (34) |
| *IgM RF+, n (%)* | 85 | (49) | 135 | (44) |
| Abbreviations: ACPA, anti-citrullinated protein antibody; CRP, C- reactive protein; DAS, disease activity score; ESR, erythrocyte sedimentation rate; IA, inflammatory arthritis; IQR, interquartile range; RA, Rheumatoid Arthritis; RF, Rheumatoid Factor; and sd, standard deviation.  * missing data for either swollen joint count or medication data (DMARDs/oral glucocorticoids) | | | | |

**Supplemental Figure S1**: ‘Quick-attained and persistent’ remission rates in (A) IgA ACPA positive vs. negative patients in the IgG ACPA positive group and in (B) IgA RF positive vs negative patients in the IgM RF positive group. ‘Quick-attained and persistent’ remission was defined as the proportion of patients that quickly attained (within 6 months) remission (DAS<1.6) and stayed in remission until 2-years of follow up. Abbreviations: ACPA, anti-citrullinated protein antibody; RF, Rheumatoid Factor.
